# Consensus on Information Provision for Patients With Early‐Stage Colorectal Cancer: A Delphi Study Among Healthcare Practitioners and Patients

**DOI:** 10.1002/cnr2.70225

**Published:** 2025-06-25

**Authors:** Ilaria Prata, Nina C. A. Vermeer, Koen C. M. J. Peeters, Fabian A. Holman, Elma Meershoek‐Klein Kranenbarg, Arwen H. Pieterse

**Affiliations:** ^1^ Department of Surgery Netherlands Cancer Institute Amsterdam the Netherlands; ^2^ Department of Surgery Leiden University Medical Center Leiden the Netherlands; ^3^ GROW, School for Oncology and Reproduction, Maastricht University Maastricht the Netherlands; ^4^ Medical Decision Making, Department of Biomedical Data Sciences Leiden University Medical Center Leiden the Netherlands

**Keywords:** consensus, Delphi, relevant patient information, surgery or intensive surveillance, T1 colorectal cancer

## Abstract

**Background:**

Patients with radically endoscopically treated T1 colorectal cancer (CRC) with at least one high‐risk histopathological characteristic are presented with the choice between additional surgical resection with lymphadenectomy or intensive surveillance. Healthcare practitioners (HCPs) from various disciplines provide information on the complex trade‐offs involved.

**Aim:**

We aimed to reach consensus on what information patients should be offered at the time of decision making.

**Methods:**

We invited HCPs and patients with early‐stage (pT1‐3N0M0) CRC to participate in a three‐round online Delphi study. In the first round, participants were asked to indicate the relevance of 163 items regarding CRC surgery and intensive surveillance, using five‐point Likert‐type scales. The following rounds only included the items on which no consensus had been reached yet, supplemented with new items that participants had suggested in the previous round(s). Criteria for consensus were defined in advance.

**Results:**

Thirty percent (109/341) of the invited participants completed ≥ 50% of items in the first round. After the third round, consensus was reached on 80/154 items regarding colon cancer and 129/179 items regarding rectal cancer; of these, respectively, 40 and 47 items were considered relevant. HCPs tended to consider more frequently occurring complications relevant compared to patients. Patients also considered rare complications relevant but expressed worries about information overload. There was clear consensus on items regarding different types of surgery and recovery expectations, the risk of anastomotic leakage and of receiving a stoma, and the risk of recurrence after both surgery and intensive surveillance.

**Conclusion:**

A consensus‐based, standardized set of information items was defined in order to facilitate that patients receive complete information in a uniform way. The results of this study aim to support patients and their HCPs to make a well‐informed decision between additional surgical resection with lymphadenectomy and intensive surveillance.

## 1 Introduction

With the introduction of nation‐wide screening for colorectal cancer (CRC) in 2014 in the Netherlands, the proportion of stage I CRC has been increasing from 18% of all diagnoses of CRC in 2010 to 31% in 2021, with stage I CRC being the most common at diagnosis [1, 2, 3, 4]. For decades, formal oncologic surgery has been the cornerstone of treatment, including patients with T1 CRC. Due to advances in the field of therapeutic endoscopy, local endoscopic resection has become an important alternative. Specific histopathological features of the lesion are associated with the risk of residual disease. Endoscopic resection is considered to be curative when all these unfavorable histopathological features are absent [5]. If one of these high‐risk features is present in the specimen after endoscopic resection, completion surgery (resection of the bowel and surrounding lymph nodes) is recommended. However, it is quite difficult to assess whether the oncological benefits of excision of potential lymph nodes and possible residual cancer tissue outweigh the risks of additional surgery. In particular, the risk of cancer recurrence after endoscopic resection and intensive surveillance ranges between 0.7% and 7.0% depending on the presence of high‐risk features [2]. Patients undergoing completion surgery for a high‐risk T1 CRC have a risk of cancer recurrence ranging between 3% and 8% [6, 7]. In addition, surgery for T1 CRC is associated with a 12.6% complication rate and postoperative mortality in 1.7% of patients [8].

Moreover, general health conditions and, increasingly, individual patients' preferences play a role in establishing the most appropriate treatment strategy for each patient. Therefore, for patients with endoscopically treated high‐risk pT1 CRC, intensive surveillance is an alternative treatment option for completion surgery. Patients are being monitored with imaging modalities, and salvage surgery is proposed in case of locoregional or distant metastases.

The two management strategies have different advantages and disadvantages with regard to oncological outcomes, risk of procedure‐related complications, and impact on quality of life. Dutch guidelines recommend that advantages and disadvantages of the different management strategies are discussed with patients in order to facilitate shared decision making [9]. However, to the best of our knowledge, there is no consensus on the extent and type of information that should be provided [10]. The aim of this study was to reach consensus on what information patients with high‐risk pT1 CRC should be offered at the time they have to make a decision between surgical resection with lymphadenectomy or intensive surveillance.

## 2 Methods

This Delphi consensus study consisted of three rounds of online questionnaires [11, 12]. The Delphi is an established method, which allows for building consensus among experts. Consensus is achieved through one or more rounds of questionnaires asking for the experts' opinion. Each next round only presents items on which consensus has not yet been reached and includes feedback on previous answers from the participants, to invite each to take others' opinions into account while responding to the items [13, 14]. The study has been approved by the joint Ethical Committee for medical centers in Leiden (Leiden University Medical Centre, LUMC), the Hague and Delft, The Netherlands (number P22.085).

### 2.1 Participants and Recruitment

Expert participants in the study were recruited among two main groups: healthcare practitioners (HCPs) and patients who were treated for early‐stage CRC. The HCPs group consisted of gastroenterologists, surgeons, and nurse specialists with experience in the care of CRC patients. They were recruited through the Dutch T1 CRC Working Group [15]. Additional presentations were done at meetings for nurse specialists.

**FIGURE 1:**
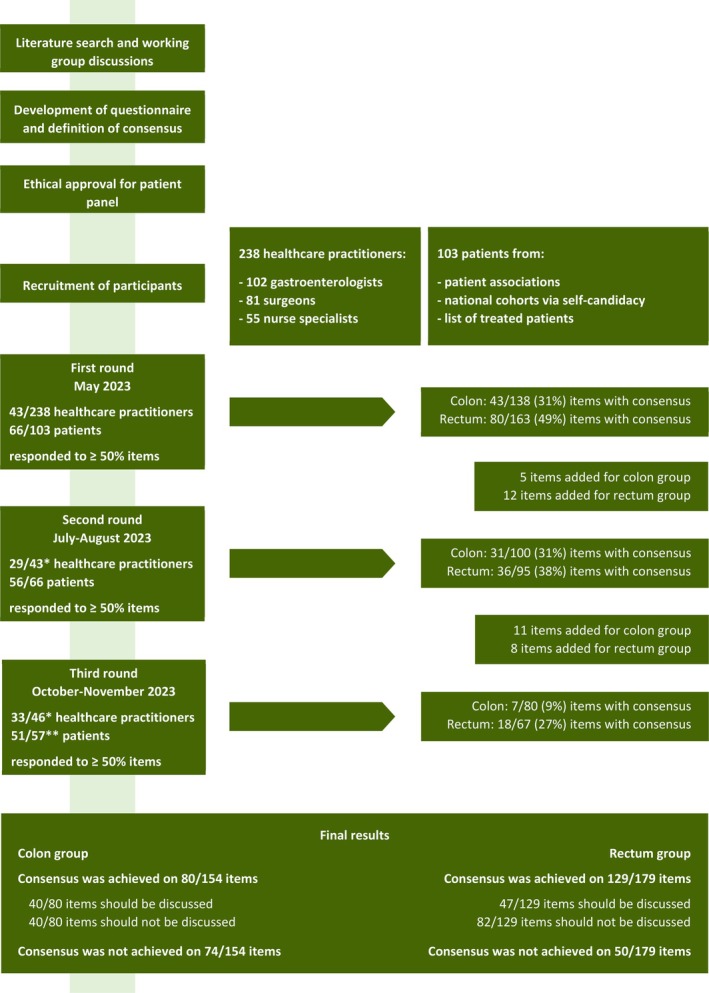
Flow chart of the study process. * In the second round all healthcare practitioners who had responded to at least 50% of all proposed items in the first round were invited. In order to maximize participation within the panel, all healthcare practitioner who had responded to at least 25% of all proposed items in the first round were invited to the third round. ** Some patients no longer desired to be invited to respond to the questionnaire.

Patients were selected if they had been treated for early‐stage CRC with surgery or local endoscopic resection followed by intensive follow‐up, between 2019 and 2022. In order to maximize patient participation, patients with a pT1‐3N0M0 cancer diagnosis were invited. Patients undergoing elective bowel resection for pT1 CRC have similar risks for surgical complications as patients with pT2‐3 disease [8]. Patient contact information was collected through a national association for patients with CRC (Stichting Darmkanker [16]), from the LUMC patient lists, and with an open invitation to participants in the Prospective National Cohort for CRC [17]. For all participants, the e‐mail address was the only personal information known to the research team. Written informed consent was obtained from all participants. The process of study design and participant recruitment is shown in Figure 1.

### 2.2 Delphi Questionnaires

The research team developed a literature and expertise‐based list of 163 items regarding the two management strategies, divided in seven sections. The first section was only included in the first questionnaire and included items referring to clinical expertise (HCPs) and educational level, diagnosis and treatment (patients). General aspects and possible complications related to surgery were divided in four sections, and one section included aspects related to intensive surveillance. We intentionally did not inform participants about how often complications occur, in order to limit study burden. The last section was an open text field that participants could use to suggest new items, in line with the principles of the Delphi approach [18]. The proposed items that the research team deemed new compared to the items already included were added in the following round(s). For patients with colon cancer, only 138 of the 163 items were relevant.

**FIGURE 2:**
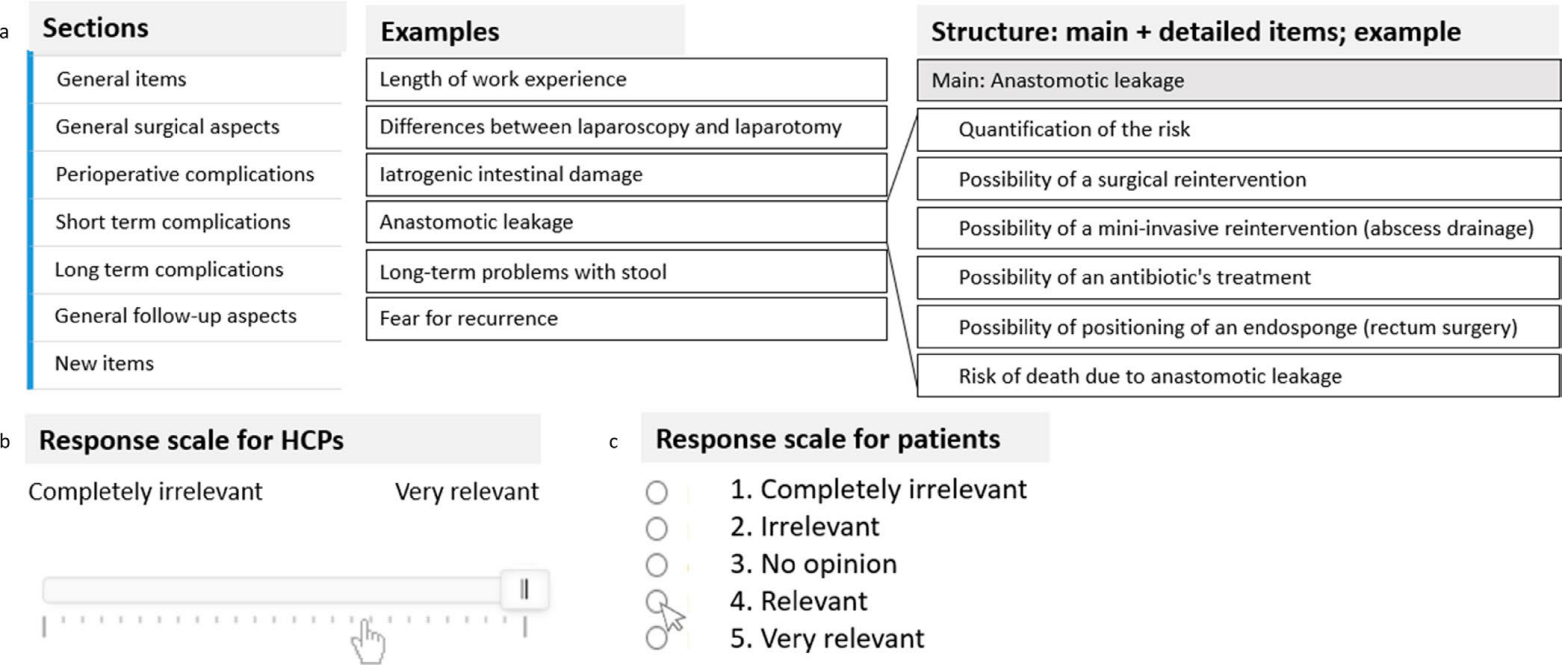
(a) General structure of the questionnaires, with example items per section; (b) Example of the structure with one main item and several detailed items. (c) Response scales for healthcare practitioners (HCPs) and patients, respectively.

For all items, participants were asked to report how relevant they found each item in the context of discussing the two management strategies during an outpatient visit, using a Likert‐type scale ranging from 1 (“Completely irrelevant”) to 5 (“Very relevant”). The content of the questionnaire was identical for the HCP and patient panels. However, items for the patient panel were formulated avoiding medical and technical terms to minimize misinterpretation of the information. Most items were presented following a recurrent structure. First, the subject was named in the “main item.” Next, more “detailed items” on the same subject became visible only if the participant did not rate it as being “completely irrelevant.” The questionnaire structure with examples of items as presented to the HCP panel is shown in Figure 2.

In all three rounds, HCPs were invited to respond to items that were relevant for all patients (regarding colon as well as rectal cancer). Patients were invited to respond only to the items relevant to their own diagnosis as some items were specific for rectal cancer surgery (e.g., complication regarding miction or sexuality).

### 2.3 Data Collection

All questionnaires were sent online via email. Castor EDC (Electronic Data Capture) software was used to create, send, and complete the questionnaires. All participants responded anonymously to each questionnaire. The participants were given 4 weeks to respond, with a reminder sent after 2 weeks. Participants were only invited to a next round if they had responded to at least 50% of the items in the previous round. However, all given votes have been taken into consideration for analysis. Moreover, we decided to extend the invitation to the third round to all HCPs who had responded to at least 25% of the items in the first round, in order to maximize their number.

### 2.4 Data Analysis and Interpretation on Results

Consensus was measured separately for both panels. In the calculation, more weight was given to the patients' opinions. Specifically, information was considered relevant to provide to patients when: (1) ≥ 70% of the participants in each panel indicated that the item was (very) relevant (score 4 or 5) or (2) between 50% and 70% of the HCPs and ≥ 70% of the patients indicated it to be (very) relevant. Consensus that information could be discarded when informing patients was considered to be reached when < 70% of the HCPs AND < 50% of the patients indicated it to be (very) relevant (score 4 or 5). In the next round, participants were informed about the items on which consensus had been reached and were invited to rate items on which consensus had not yet been reached.

## 3 Results

We invited 238 HCPs and 103 patients to participate. In total, 43 (18%) HCPs and 66 (64%) patients responded to a minimum of 50% of the items in the first round. In the second round, these numbers were 29 (12%) and 56 (54%) respectively, and in the third round, 33 (14%) and 51 (50%). Overall, no clear differences were seen in how gastroenterologists, surgeons, and nurse specialists voted. Of the 43 HCPs who responded to at least 50% of items in the first round, 14 (33%) were gastroenterologists, 19 (44%) were surgeons, and 10 (23%) were nurse specialists. Most (79%) had more than 5 years of experience with treating CRC patients, and the majority (63%) reported encountering five to fifteen CRC patients each month (Table 1). Among the 66 patients who responded to the items in section 1, the majority (58%) were male and were between 60 and 69 years at diagnosis (39%). More than half of the patients (58%) had achieved a high level of education, defined as at least a university bachelor's degree (Table 2).

**TABLE 1:** Healthcare practitioners' work‐related characteristics^a^.

General items—Section 1	Responders *n*/*N* (%)
Function	
Gastroenterologist	14/43 (33)
Surgeon	19/43 (44)
Nurse‐specialist	10/43 (22)
Years of experience	
< 1	2/43 (5)
1–3	1/43 (2)
3–5	6/43 (14)
5–10	18/43 (42)
> 10	16/43 (37)
Frequency of discussions per month	
< 5	6/43 (14)
5–15	27/43 (63)
15–25	8/43 (19)
> 25	2/43 (5)
Proportion of T1 CRC patients discussed in multidisciplinary meetings^b^	
< 25%	2/43 (5)
25%–50%	1/43 (2)
50%–75%	2/43 (5)
> 75%	5/43 (12)
100%	33/43 (77)

^a^ Data regarding healthcare practitioners who responded to at least 50% of the items in the first round are presented.

^b^ The proportion is indicated by healthcare practitioners based on common practice in their institution.

**TABLE 2:** Patients' sociodemographic, diagnosis, and treatment‐related characteristics^a^.

General items—Section 1	Responders *n*/*N*
Gender	
Male	38/66 (58)
Female	28/66 (42)
Age at diagnosis	
40–49	2/66 (3)
50–59	17/66 (26)
60–69	26/66 (39)
70–79	19/66 (29)
≥ 80	2/66 (3)
Highest education^a^	
Lower	9/66 (14)
Intermediate	19/66 (29)
Higher	38/66 (58)
Tumor location	
Colon	40/66 (61)
Rectum	26/66 (39)
Type of treatment	
Surgical resection	61/66 (92)
No surgery and intensive surveillance	5/66 (8)
Surgical complications	
Yes, within 3 days after operation	17/61 (28)
Yes, more than 3 days after operation	15/61 (25)
No complications	25/61 (41)
Unknown	4/61 (7)
Satisfied with information^b^	
Yes	5/66 (8)
No	54/66 (82)
Unknown	7/66 (11)

^a^ Data regarding patients who responded to at least 50% of the items in the first round are presented.

^b^ Patients were asked whether they perceive they have received sufficient information on the two management strategies.

### 3.1 First Round

The first questionnaire consisted of 138 items regarding colon cancer and 163 items regarding rectal cancer. For colon cancer, both panels considered 35 (25%) items to be relevant, 8 (6%) not relevant, and on 95 items (69%) no consensus was reached. For rectal cancer, the panels considered 40 (25%) items to be relevant, 40 (25%) items not relevant, and no consensus was reached on 83 items (51%). Based on the participants' suggestions, 12 new items were added to the questionnaire, of which only five were pertinent to both patients with colon and rectal cancer.

### 3.2 Second Round

The participants were invited to respond to 100 and 95 items regarding colon and rectal cancer, respectively. For colon cancer, consensus was reached on 31 (31%) additional items: three (3%) were considered relevant and 28 (28%) not relevant. For rectal cancer, consensus was reached on 36 (38%) items: four (4%) were judged relevant and 32 (34%) not relevant. By the end of the second round, no consensus had yet been reached on 69 (69%) items for colon cancer and 59 (62%) items for rectal cancer. After the second round, 11 new items were added to the questionnaire, of which eight were relevant for both patients with colon and rectal cancer. Along with suggesting new items, many patients used the open text field or sent an email to the researcher to share their thoughts. Multiple patients expressed their worries regarding the severity and abundance of possible complications, stating that they probably would have preferred to avoid surgery and/or they would have been overwhelmed with the possible risks if all the items had been mentioned to them during the clinical encounter.

### 3.3 Third Round

The participants were invited to respond to 80 and 67 items regarding colon and rectal cancer, respectively. For colon cancer, consensus was reached on seven items (9%), three (4%) being relevant and four (5%) not relevant. For rectal cancer, consensus was reached on 18 (27%) items, of which three (4%) were considered to be relevant and 15 (22%) were deemed not relevant. At the end of the third round, no consensus had been reached on 74/154 (48%) items for colon cancer and 50/179 (28%) items for rectal cancer.

### 3.4 Relevant Items

#### 3.4.1 General Items on Surgery

The items that both panels found relevant to discuss with patients with early‐stage CRC are presented in Table 3; of these, a selection of items on which agreement was highest is listed in Table 4. The panels considered information about the difference between laparoscopic and open surgery and the possibility of conversion to be relevant both for patients with colon and rectal cancer; for the latter, also information about differences between a Low Anterior and Abdominoperineal resection is relevant. Additionally, the expected duration of admission and the possibility of quickly resuming a normal diet and light physical activity were considered relevant. Finally, the risk and possible location of tumor recurrence and the planned follow‐up for early detection of possible recurrences should be discussed.

**TABLE 3:** Items on which participating healthcare practitioners and patients reached consensus on whether they should be discussed with all patients.

Item	Colon	Rectum
General items on surgery		
Differences between laparotomy of laparoscopy	Yes	Yes
Differences in terms of duration of admission after laparotomy or laparoscopy	Yes	Yes
Possibility of conversion from laparoscopy to laparotomy during surgery	Yes	Yes
Differences between low anterior‐ and abdominoperineal resection	NA	Yes
General aspects of the ERAS protocol (enhanced recovery after surgery)	Yes	Yes
Possibility to reintroduce normal diet in first days postoperatively according to ERAS protocol	Yes	Yes
Possibility to receive physiotherapy and encouragement to walk in first days postoperatively according to ERAS protocol	Yes	Yes
Expected duration of hospitalization after surgery	Yes	Yes
Risk of recurrence after surgery	Yes	Yes
Quantification of the risk of tumor recurrence after surgery	Yes	NC
Location of possible tumor recurrences after surgery	Yes	Yes
Program of follow‐up after surgery	Yes	Yes
Frequency of follow‐up encounters after surgery	Yes	Yes
Type of follow‐up investigations after surgery	Yes	Yes
Life expectancy after surgery	Yes	Yes
Perioperative complications		
Risk of abdominal bleeding during surgery	Yes	Yes
Short‐term postoperative complications (< 3 months after surgery)		
Anastomotic leakage	Yes	Yes
Quantification of the risk of anastomotic leakage	Yes	NC
Possibility of a surgical reintervention	Yes	Yes
Possibility of a mini‐invasive reintervention (abscess drainage)	Yes	No
Risk of death due to anastomotic leakage	Yes	No
Possibility to place a stoma during surgery	Yes	Yes
Quantification of the risk of placement of stoma	Yes	Yes
Risk of complications relating to the stoma	Yes	Yes
Risk of infection of the surgical wound	Yes	Yes
Post‐operative abdominal bleeding	Yes	No
Post‐operative bowel complications	Yes	Yes
Changes in faecal consistency	Yes	Yes
Faecal urgency	Yes	Yes
Increased stool frequency	Yes	NC
Clustering of stools	NC	Yes
Faecal incontinence	Yes	Yes
Abdominal pain after surgery	Yes	Yes
Deterioration of physical condition (in the short term)	Yes	Yes
Long‐term postoperative complications (> 3 months after surgery)		
Deterioration of physical condition (in the long term)	Yes	Yes
Long‐term bowel complications	NC	Yes
Change of faecal consistency	NC	Yes
Clustering of stools	NC	Yes
Faecal incontinence	NC	Yes
Long‐term problems with miction	NA	Yes
Sexual dysfunctions	NA	Yes
Erection dysfunctions	NA	Yes
Retrograde ejaculation	NA	Yes
Dyspareunia	NA	Yes
Vaginal dryness	NA	Yes
Intensive surveillance		
Characteristics of follow‐up	Yes	Yes
Risk of tumor recurrence	Yes	Yes
Risk of distant metastases	Yes	Yes
Fear of tumor recurrence	Yes	Yes
Necessity of a second operation in case of tumor recurrence	Yes	Yes
Possible non‐surgical treatments in case of tumor recurrence	Yes	Yes
Risk that no curative treatment is possible in case of tumor recurrence	Yes	Yes

Abbreviations: NA = not applicable; NC = no consensus.

**TABLE 4:** Items on which the highest agreement was reached, voting round, and proportion of participants in each panel who reported the item to be (very) relevant.

Item	Voting colon cancer	Voting rectal cancer
General items on surgery		
Possibility of conversion from laparoscopy to laparotomy during the operation	At 1st round HCP 81%, *PCC* 82%	At 1st round HCP 81%, *PRC* 88%
Differences between Low Anterior‐ and Abdominoperineal Resection	NA	At 1st round HCP 81%, *PRC* 88%
Expected duration of hospitalization after surgery	At 1st round HCP 83%, *PCC* 92%	At 1st round HCP 83%, *PRC* 80%.
Risk of tumour recurrence after surgery	At 1st round HCP 72%, *PCC* 100%	At 1st round HCP 72%, *PRC* 100%.
Program of follow‐up after surgery	At 1st round HCP 72%, *PCC* 100%	At 1st round HCP 72%, *PRC* 100%.
Short‐term postoperative complications (< 3 months after surgery)		
Anastomotic leakage	At 1st round HCP 100%, *PCC* 92%	At 1st round HCP 100%, *PRC* 80%.
Quantification of risk of anastomotic leakage	At 2nd round HCP 90%, *PCC* 77%	NC
Necessity of second operation for correction of anastomotic leakage	At 1st round HCP 93%, *PCC* 92%	At 1st round HCP 93%, *PRC* 72%
Possibility to place a stoma during surgery	At 1st round HCP 100%, *PCC* 100%	At 1st round HCP 100%, *PRC* 96%.
Post‐operative bowel complications	At 1st round HCP 91%, *PCC* 73%	At 1st round HCP 98%, *PRC* 80%.
Faecal incontinence^a^	At 1st round HCP 52%, *PCC* 91%	At 1st round HCP 88%, *PRC* 87%.
Intensive surveillance		
Characteristics of follow‐up	At 1st round HCP 85%, *PCC* 97%	At 1st round HCP 85%, *PRC* 100%.
Risk of tumor recurrence	At 2nd round HCP 78%, *PCC* 91%	At 1st round HCP 78%, *PRC* 86%.
Risk that no curative treatment is possible in case of tumor recurrence	At 1st round HCP 76%, *PCC* 93%	At 1st round HCP 76%, *PRC* 91%.

Abbreviations: HCP = healthcare practitioners; NA = not applicable; NC = no consensus; PCC = patients with colon cancer; PRC = patients with rectal cancer.

^a^ Consensus was achieved for both colon and rectal cancer; however, this item only reached high consensus for patients with rectal cancer.

#### 3.4.2 Perioperative and Short‐Term Complications of Surgery

Of the possible complications that could occur during surgery, only the risk of intraoperative bleeding was considered relevant for both types of cancer. Anastomotic leakage and the probability of receiving a stoma were considered relevant after the first round by all HCPs and by over 80% of patients in both groups. Also, the risk of wound infection, post‐operative bowel complications, abdominal pain, and deterioration of physical condition in the short term should be discussed with all patients. Post‐operative abdominal internal bleeding was considered relevant for patients with colon cancer only. Finally, the risk of urinary dysfunctions in the short term was considered relevant for patients with rectal cancer, as this complication mainly occurs after rectal surgery.

#### 3.4.3 Long‐Term Complications of Surgery

The risk of deterioration of physical condition in the long term should be discussed both with patients with colon and rectal cancer. Moreover, patients with rectal cancer should be informed about the risk of long‐term dysfunctions regarding stool, miction, and sexuality. The panels considered the majority of items quantifying risks (9/15, 60% and 14/18, 78% for colon and rectal cancer, respectively) and the probabilities of dying as a consequence of various complications (5/9, 56% for colon and 8/9, 89% for rectal cancer) as not relevant.

#### 3.4.4 Surveillance

Recommendations on which items should be discussed regarding the choice of not operating after endoscopic resection were the same for patients with both colon and rectal cancer. The panels recommended discussing the risk of tumor recurrence, both as local tumor regrowth and as distant metastasis. On the same subject, the possible anxiety connected with the risk of tumor recurrence, the type and schedule of the necessarily strict follow‐up, and the possible surgical or conservative salvage treatments should also be discussed with patients.

#### 3.4.5 Major Differences Between HCPs and Patients

**FIGURE 3:**
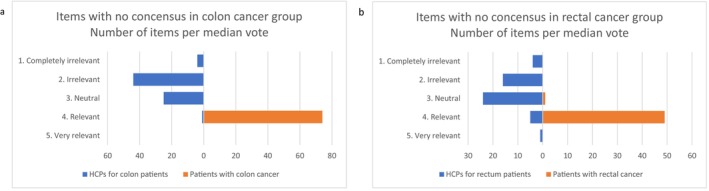
Overview of the discrepancies between HCPs and patients relative to the items on which no consensus has been reached. The median of all votes given in the third round to these items are shown. Patients considered more items to be relevant than HCPs, both regarding colon cancer (a) and rectal cancer (b).

HCPs considered the majority of items referring to anastomotic leakage relevant. Similarly, they found that bowel complications should be discussed with patients with rectal cancer. Most HCPs considered other items on this topic irrelevant (median of votes = 2). In contrast, at least half of the patients considered the majority of items on which no consensus was found relevant (median of votes = 4), regardless of the type of cancer. While consensus was reached on the majority of “main items” (e.g., “anastomotic leakage,” “stoma complications,” and “bowel complications”), no consensus was reached on most of the “detailed items” (58% of all items with no consensus regarding colon cancer and 68% regarding rectal cancer). The median of votes given by each panel to the items on which no consensus was reached at the end of the study is presented in Figure 3. The exact items on which the panels did not reach consensus, with the corresponding median of votes per panel, are presented in Figure 1 in the Tables A1 and A2.

## 4 Discussion

After three rounds, HCPs and patients reached consensus on the majority of items regarding the characteristics of the two management strategies for colon and rectal cancer. Forty information items are considered relevant regarding colon cancer, and forty‐seven items regarding rectal cancer. Both HCPs and patients considered items regarding the surgical technique and the period of post‐operative hospitalization relevant. Also, HCPs and patients wished to discuss the risk of tumor recurrence in case of surgery or surveillance. Anastomotic leakage, the possibility of placing a stoma, and experiencing post‐operative complications regarding stool, miction, and sexual function (the latter two specifically for rectal cancer surgery) appeared to be relevant to both groups. Additionally, patients stressed the importance of providing information about aspects that are less strictly clinical, such as preserving sufficient physical condition and resuming daily activities, including a regular diet. While consensus has been reached on most “main items,” the majority of the items on which no consensus has been reached referred to “detailed items.”

Compared to other Delphi studies, the overall level of consensus reached within this study seems quite low [12, 19, 20, 21, 22]. Clearly, given that we presented a high number of items (163), the absolute number of items on which consensus has been reached is still considerable (80 and 129 for colon and rectal cancer, respectively). Notably, many Delphi studies have only involved medical professionals who were asked to give their opinion on technical matters [12, 21, 23], and previous findings suggest that HCPs' and patients' opinions can substantially differ regarding the importance of information [24]. The latter prompted us to invite both HCPs involved in treating CRC patients and treated CRC patients.

Participants were not informed about the relative frequency of complications. Nevertheless, our study showed that HCPs uniformly considered it more often relevant to tell patients about complications that occur more frequently. Most participating HCPs (34/43) had more than 5 years' experience with treating CRC patients; therefore, they may have been well aware of how frequently particular complications occur.

**FIGURE A1:**
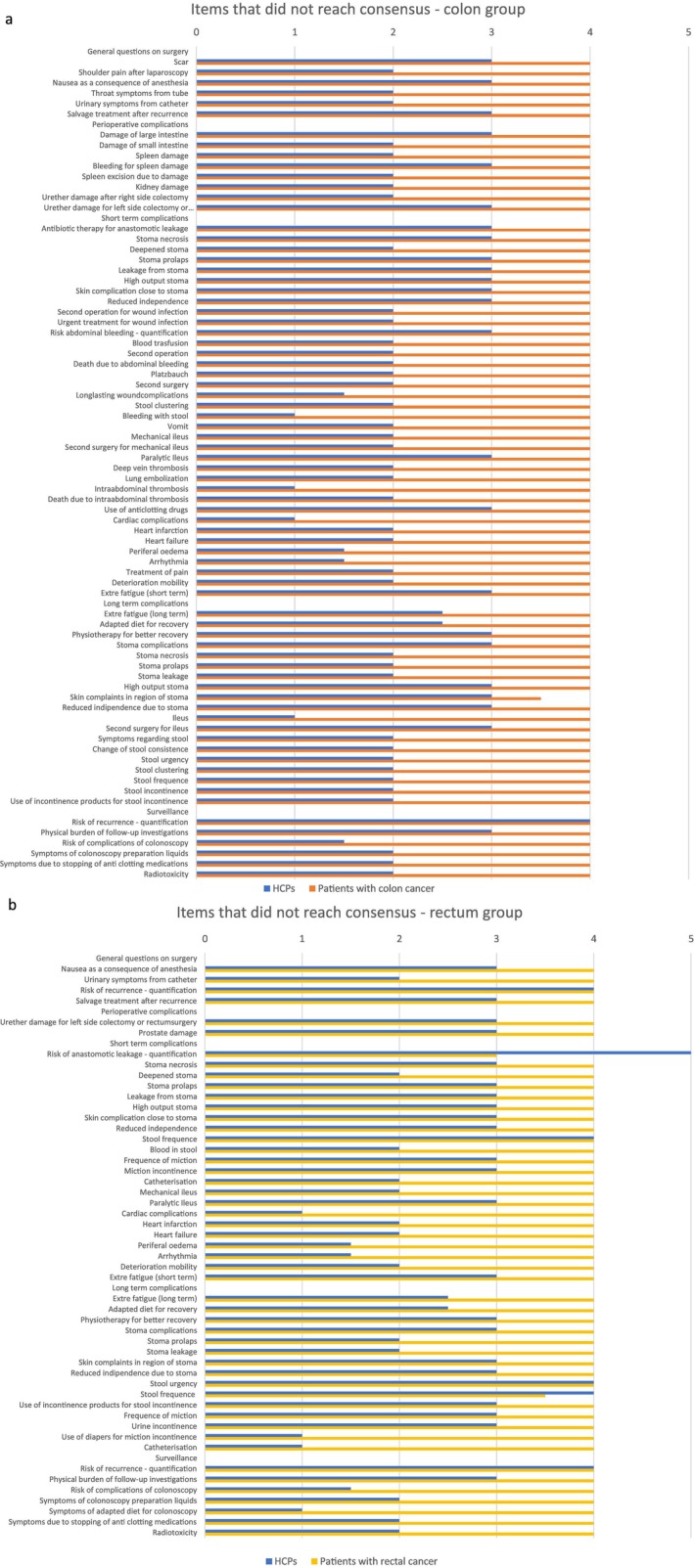
Median of the votes on the items on which no consensus has been reached in the colon (a) and rectum (b) group, by panel.

The patient panel, in contrast, considered the vast majority of items relating to surgical complications to be relevant. In particular, items referring to relatively rare complications (e.g., cardiac complications) and “detailed items” (e.g., heart infarction or failure, see Figure A1) were considered relevant. At the same time, many patients shared that they might have refrained from surgery if all possible complications had been discussed with them, as they would have felt overwhelmed by the possible risks. The patients were asked about which information they would have found relevant to receive at the time of decision making; note that they had faced the treatment decision one to 4 years before. It is known that information needs can vary across patients and over time [10, 25], with a stronger focus on diagnosis‐related information early in the course of disease and more interest in medium‐ and long‐term complications later on [10]. In fact, soon after receiving a cancer diagnosis emotional distress can strongly limit patients' ability to understand and process information about treatment benefits and harms. However, later on in the course of the disease patients often become more critical about the information they have received earlier [25] and patients can feel they have been insufficiently informed once complications occur [10]. Additionally, results from the patients' panel show a challenging balance between considering a lot of information to be relevant and the risk of becoming overwhelmed by an excess of information provided at one time point.

Comparing oncological outcomes after surgery and intensive surveillance for high‐risk T1 CRC is the subject of current research. Providing patients with complete and uniform information on treatment options could support a more effective process of shared decision making in daily clinical practice. Obviously, complete standardization is not the ultimate goal, given the considerable variation between patients in terms of demographic characteristics and non‐cancer‐related health conditions. For example, some information categories are likely more relevant for younger and fitter patients, whereas complications caused by pre‐existing cardiovascular disorders (e.g., anastomotic leakage) are more relevant to discuss with elderly and comorbid patients.

An important next step is to find ways to deliver the information clearly, in manageable amounts and in a timely manner. One suggestion to address variability in information needs and possibly to address time pressure during clinical encounters is to prepare easily accessible patient information material that patients and caregivers can consult prior to and following the consultation as input to making the decision. Ideally, patients should be able to access information according to their interests (i.e., ranging from general information about outcomes of a particular management strategy, to information about each single risk of treatment). Additionally, providing a set of standardized information in advance is expected to make consultations more efficient. A number of websites exist that offer information [26] or decision tools [27] in easily understandable Dutch regarding CRC and possible treatments depending on disease stage. Based on the consensus reached in the present study, we could consider adapting the material offered on these websites and enriching it with new easily accessible infographics or information sheets. These could be developed and made public with the support of non‐profit organizations that are largely known to Dutch patients such as Stichting Darmkanker [16].

This study has several limitations. First, the participation rate was low, especially among the HCPs (18%). One reason could be that we approached a vast number of HCPs and that not all of them had been informed about the study at the time they were approached. This may have led some to ignore the invitation. Also, we have decided to present a broad range of possible discussion topics which resulted in a high number of items (163). This could have discouraged some participants who did not complete 50% of the questionnaire and were therefore not invited for the second round. In order to increase participation among HCPs, we invited in the third round all those who had completed 25% of items in the first round. However, the absolute number of participants was in line with other Delphi studies [11, 21, 22, 28, 29]. Additionally, the vast majority of participating HCPs have more than 5 years' experience with early‐stage CRC patients. Therefore, we expect that they are representative of the opinions and experiences of a numerous group of Dutch HCPs who commonly discuss this trade‐off with patients. Second, there was little heterogeneity in how the disease of participating patients had been managed, with 61/66 patients who underwent surgery. Recent data from a Dutch national registry show that respectively 50% and 33% of patients with colon and rectal cancer and high‐risk features underwent segmental surgery after a curative endoscopic resection [30], which is different from the proportion of operated patients in the patient panel. Notably, our data fully relies on patients' responses to the questionnaire and, in order to guarantee anonymity, the study team was not informed on how patients have been treated. Ideally, we would have included more patients with a personal experience of intensive surveillance. However, we do not expect this discrepancy to jeopardize the consensus we reached with this study.

## 5 Conclusion

The results of this Delphi study aim to support patients and their HCPs to make a well‐informed decision between additional surgical resection and intensive surveillance. The consensus reached on the most relevant information can serve as a tool to provide patients with complete information in a uniform way. Moreover, this consensus study helps to overcome differences in information provision in future studies assessing the oncological safety of omitting surgery after endoscopic resection of high‐risk T1 CRC.

## Author Contributions

All authors had access to the data in the study and take responsibility for the integrity of the data and the accuracy of the data analysis. I.P., N.C.A.V., K.C.M.J.P., F.A.H., and A.H.P. created the conceptualization and methodology. I.P. performed the investigation, formal analysis, and writing – original draft. I.P., N.C.A.V., K.C.M.J.P., F.A.H., E.M.‐K.K., and A.H.P. performed the writing – review and editing. Data curation was done by E.M.‐K.K. and I.P. Supervision was done by A.H.P., K.C.M.J.P., and N.C.A.V. and funding acquisition was by I.P.

## Ethics Statement

The study has been approved by the joint Ethical Committee for medical centres in Leiden (LUMC), the Hague, and Delft (number P22.085).

## Consent

Written informed consent was obtained from all participants.

## Conflicts of Interest

The authors declare no conflicts of interest.

## Data Availability

The data collected and used for this study are original and no data or material has been requested by other sources. The data used for this study are stored on a secured drive on the servers of the Leiden University Medical Centre, Leiden, the Netherlands. Data have been managed and storage has been coordinated by the main researcher with the supervision of the local Clinical Research Centre. Access to these data is limited and can be granted to the members of the study group upon request.

## Appendix A

**TABLE A1:** Items about colon cancer that both panels considered not relevant to discuss with all patients.

Item
Short‐term complications (< 3 months after surgery)
Quantification of the risk of infection of the surgical wound
Treatment with antibiotics for infection of the surgical wound
Quantification of the risk of a platzbauch
Quantification of the risk of bowel complications
Risk of nausea
Quantification of the risk of nausea
Necessity of a nasogastric tube for nausea
Necessity of a nasogastric tube for vomit
Risk of aspiration in case of vomit
Risk of death due to aspiration
Risk of anorexia
Necessity of a feeding tube in case of anorexia
Necessity of parenteral administration of nutrients in case on anorexia
Quantification of risk of a mechanic ileus
Necessity of a nasogastric tube for a mechanic or paralytic ileus
Treatment of deep vein thrombosis
Quantification of the risk of lung embolism
Treatment of lung embolism
Death due to lung embolism
Treatment of intra‐abdominal thrombosis
Risk of pneumoniae
Quantification of the risk of pneumoniae
Death due to pneumoniae
Death due to heart complications
Long‐term complications (> 3 months after surgery)
Incisional hernia
Quantification of the risk of incisional hernia
Necessity of an elastic band to correct incisional hernia
Necessity of the surgical placement of a mesh to correct incisional hernia
Retracted stoma
Skin complaints in the area of the stoma
Quantification of the risk of ileus
Necessity of a nasogastric tube in case of ileus
Quantification of the risk of bowel complications
Intensive surveillance
Psychological discomfort related with follow‐up investigations
Risk of perforation (during colonoscopy)
Risk of bleeding (during colonoscopy)
Necessity for salvage surgery to correct complications occurred during colonoscopy
Risk of symptoms related to the adapted diet prescribed before a colonoscopy
Death due to complications of a colonoscopy

**TABLE A2:** Items about rectal cancer that both panels considered not relevant to discuss with all patients.

Item
General questions on surgery
Differences in the size of the scar after laparotomy versus laparoscopy
Risk of experiencing shoulder pain after laparoscopy
Risk of experiencing throat discomfort after intubation for general anesthesia
Perioperative complications
Risk of damaging the large intestine
Risk of damaging the small intestine
Risk of damaging the spleen
Short‐term complications (< 3 months after surgery)
Risk of abscess in area of anastomotic leakage
Possible treatment with antibiotics in case of anastomotic leakage
Possible use of an endosponge as a treatment for anastomotic leakage
Death due to anastomotic leakage
Quantification of the risk of infection of the surgical wound
Possible second surgery in case of infection of the surgical wound
Possible urgency for surgical treatment in case of infection of the surgical wound
Possible treatment with antibiotics in case of infection of the surgical wound
Risk of infection of the surgical wound after an Abdominoperineal Resection
Risk of dehiscence of the surgical wound after an Abdominoperineal Resection
Post operatory abdominal bleeding (after surgery)
Quantification of the risk of abdominal bleeding
Possible blood transfusion in case of abdominal bleeding
Possible second surgery in case of abdominal bleeding
Death in case of abdominal bleeding
Risk of a platzbauch
Quantification of the risk of a platzbauch
Possible second surgery in case of a platzbauch
Risk of long‐lasting wound complications in case of a platzbauch
Quantification of the risk of bowel complications
Quantification of the risk of difficulties with miction
Increased urgency for miction
Need of diapers for miction incontinence
Risk of nausea
Quantification of the risk of nausea
Necessity of a nasogastric tube for nausea
Risk of vomiting
Necessity of a nasogastric tube for vomiting
Risk of aspiration in case of vomiting
Death due to aspiration
Risk of anorexia
Necessity of a feeding tube in case of anorexia
Necessity of parenteral administration of nutrients in case on anorexia
Quantification of risk of a mechanic ileus
Necessity of a nasogastric tube for a mechanic or paralytic ileus
Possible surgical correction of mechanic ileus
Risk of deep vein thrombosis
Treatment of deep vein thrombosis
Risk of lung embolization
Quantification of the risk of lung embolism
Treatment of lung embolism
Death due to lung embolism
Risk of intra‐abdominal thrombosis
Treatment of intra‐abdominal thrombosis
Death due to intra‐abdominal thrombosis
Possible use of anticlotting medication
Risk of pneumoniae
Quantification of the risk of pneumoniae
Death due to pneumoniae
Death due to heart complications
Treatment of postoperative abdominal pain
Long‐term complications (> 3 months after surgery)
Incisional hernia
Quantification of the risk of incisional hernia
Necessity of an elastic band to correct incisional hernia
Necessity of the surgical placement of a mesh to correct incisional hernia
Necrosis of stoma tissue
Retracted stoma
High output stoma
Risk of ileus (on the long term)
Quantification of the risk of ileus (on the long term)
Possible second surgery in case of ileus (on the long term)
Necessity of a nasogastric tube in case of ileus
Quantification of the risk of bowel complications
Quantification of the risk of problems with miction
Urgency for miction
Quantification of the risk of sexual dysfunctions
Perineal hernia after an Abdominoperineal Resection
Sitting discomfort after an Abdominoperineal Resection
Intensive surveillance
Psychological discomfort related to follow‐up investigations
Risk of perforation (during colonoscopy)
Risk of bleeding (during colonoscopy)
Necessity for salvage surgery to correct complications occurred during colonoscopy
Death due to complications of a colonoscopy
